# Primary Parosteal Osteosarcoma of the Bone With Rhabdomyosarcomatous Features: A Rare Bone Tumour With a Pathological Dilemma

**DOI:** 10.7759/cureus.109196

**Published:** 2026-05-19

**Authors:** Ningthoujam D Devi, Vikas K Jagtap, Daffilyne L Nongrum, Anthialisha Nongkynrih, Emihaka Warjri

**Affiliations:** 1 Radiation Oncology, North Eastern Indira Gandhi Regional Institute of Health and Medical Sciences (NEIGRIHMS), Shillong, IND; 2 Pathology, Civil Hospital, Shillong, IND

**Keywords:** dedifferentiation, osteosarcoma, parosteal osteosarcoma, rare tumour, rhabdomyosarcoma

## Abstract

Primary osteosarcoma of the bone with rhabdomyosarcomatous characteristics constitutes a remarkably uncommon and distinct clinicopathological entity. This tumour exhibits a dual histological pattern, incorporating conventional osteosarcomatous morphology alongside rhabdomyosarcomatous cells, making diagnosis and management particularly challenging. We present a case of a 45-year-old woman with a progressively increasing painful mass in the proximal part of the leg. Radiological and histopathological investigations confirmed proximal tibial parosteal osteosarcoma exhibiting rhabdomyosarcomatous differentiation. The patient was treated with a multimodality approach involving above-knee amputation surgery followed by adjuvant chemotherapy. This clinical scenario highlights the importance of recognizing uncommon histological variants of osteosarcoma, as well as the future needs of additional research to develop standardized treatment algorithms and reliable prognostic indicators for this rare tumour.

## Introduction

Osteosarcoma is a mesenchymal neoplasm that constitutes approximately 20% of all primary bone tumours [1]. Osteosarcoma develops from primitive bone-forming mesenchymal cells and is a significant entity in the spectrum of skeletal malignancies. Primary osteosarcoma represents an aggressive bone malignancy characterized by a unique bimodal age incidence pattern. This neoplasm predominantly affects adolescents and older adults, especially individuals with Paget's disease of the bone. The long bones represent the most frequent anatomical location for this tumour [2]. According to the World Health Organization (WHO) classification system, osteosarcoma is categorized into eight histological and anatomical variants: conventional, secondary, telangiectatic, small-cell, low-grade, parosteal, periosteal, and high-grade surface variants. These categories can be further classified based on the primary matrix composition within the tumour, yielding osteoblastic, chondroblastic or fibroblastic subtypes [3]. Rhabdomyosarcoma is a type of soft tissue sarcoma tumour of immature skeletal muscle cells, i.e., rhabdomyoblasts. As per literature, several tumours have shown rhabdomyosarcomatous differentiation, which worsens the prognosis including the malignant Triton tumour, which is a malignant peripheral nerve sheath tumour with rhabdomyosarcomatous differentiation, solitary fibrous tumours, carcinosarcoma, cystosarcoma phylloides [4-7]. 

Parosteal osteosarcoma is a low-grade osteogenic sarcoma showing a low-grade and low cellular spindle cell component with less frequent mitosis interspersed with woven and lamellar bone trabeculae [8]. Dedifferentiation occurs in 16 to 24 % of cases [8]. With dedifferentiation, the prognosis of the tumours will be similar to that of a conventional osteogenic sarcoma. The dedifferentiation may appear like high-grade osteosarcoma, undifferentiated spindle cell sarcoma, chondroblastoma, and rarely rhabdomyosarcoma. Primary osteosarcoma with rhabdomyosarcomatous differentiation is an extremely rare malignant bone neoplasm. The exact incidence of this specific variant is not well established in the literature due to its rarity. The dual histopathological presentation of this rare bone malignancy sets it apart as a unique clinicopathological entity within the spectrum of osseous neoplasms [9]. This unusual association warrants further investigation in medical research. In this report, we present a case of parosteal osteosarcoma exhibiting rhabdomyosarcomatous differentiation.

## Case presentation

**Figure 1:**
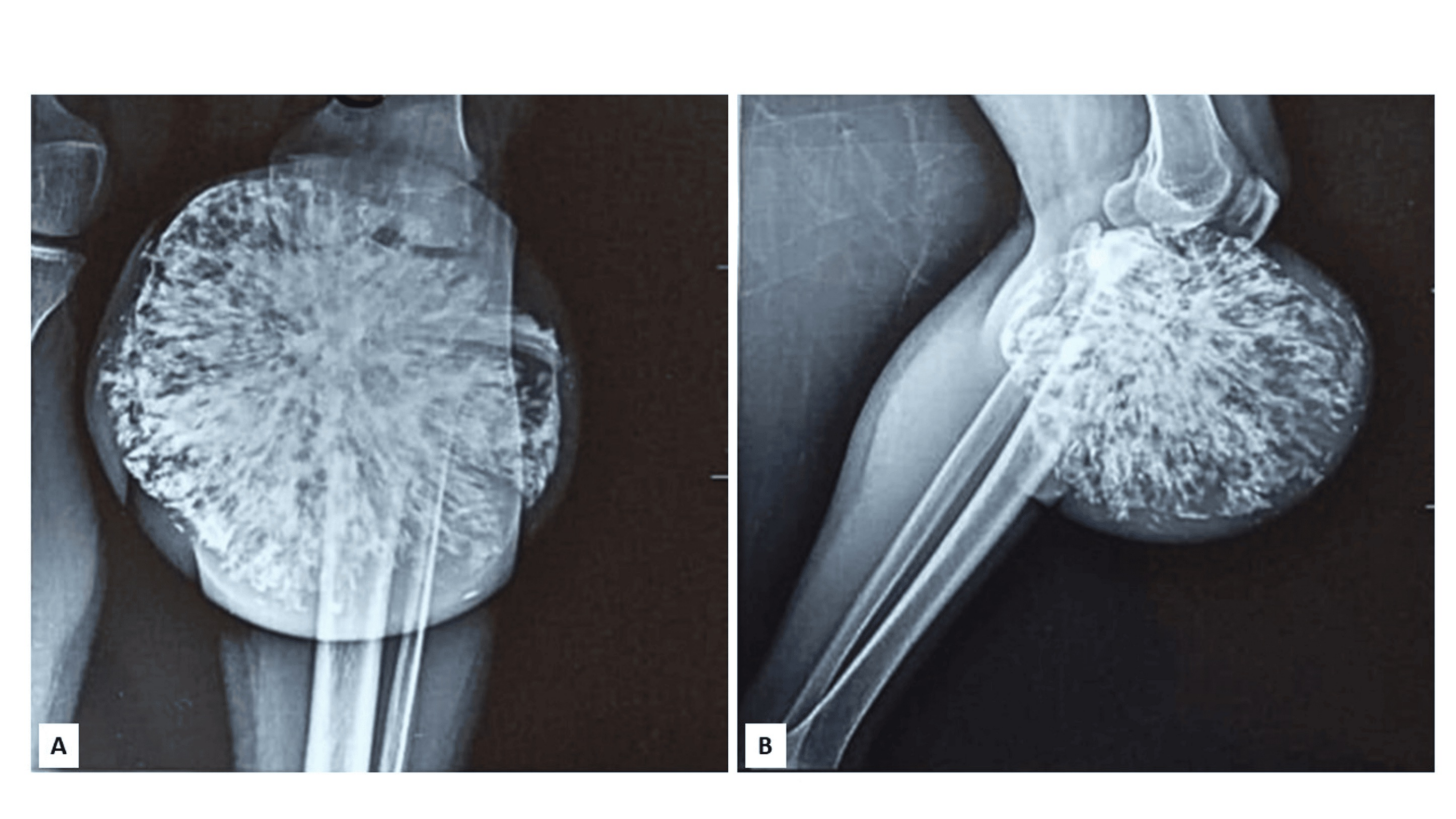
X-ray showing antero-posterior (A) and lateral (B) views of the left knee with a large radiolucent area with radiating calcific densities extending from the center (typical sunburst or sunray appearance)

In October 2024, a 45-year-old woman sought medical attention after experiencing progressive pain and swelling in her left leg for the past six months. The patient reported no constitutional symptoms like fever or weight loss during the clinical evaluation. During the physical examination, a tender and firm mass, approximately 10×15 cm in size, was noted in the left knee area. X-ray of the left knee revealed a radiolucent lesion with radiating calcific densities within the epi-metaphyseal region of the proximal tibia (Figure 1).

**Figure 2:**
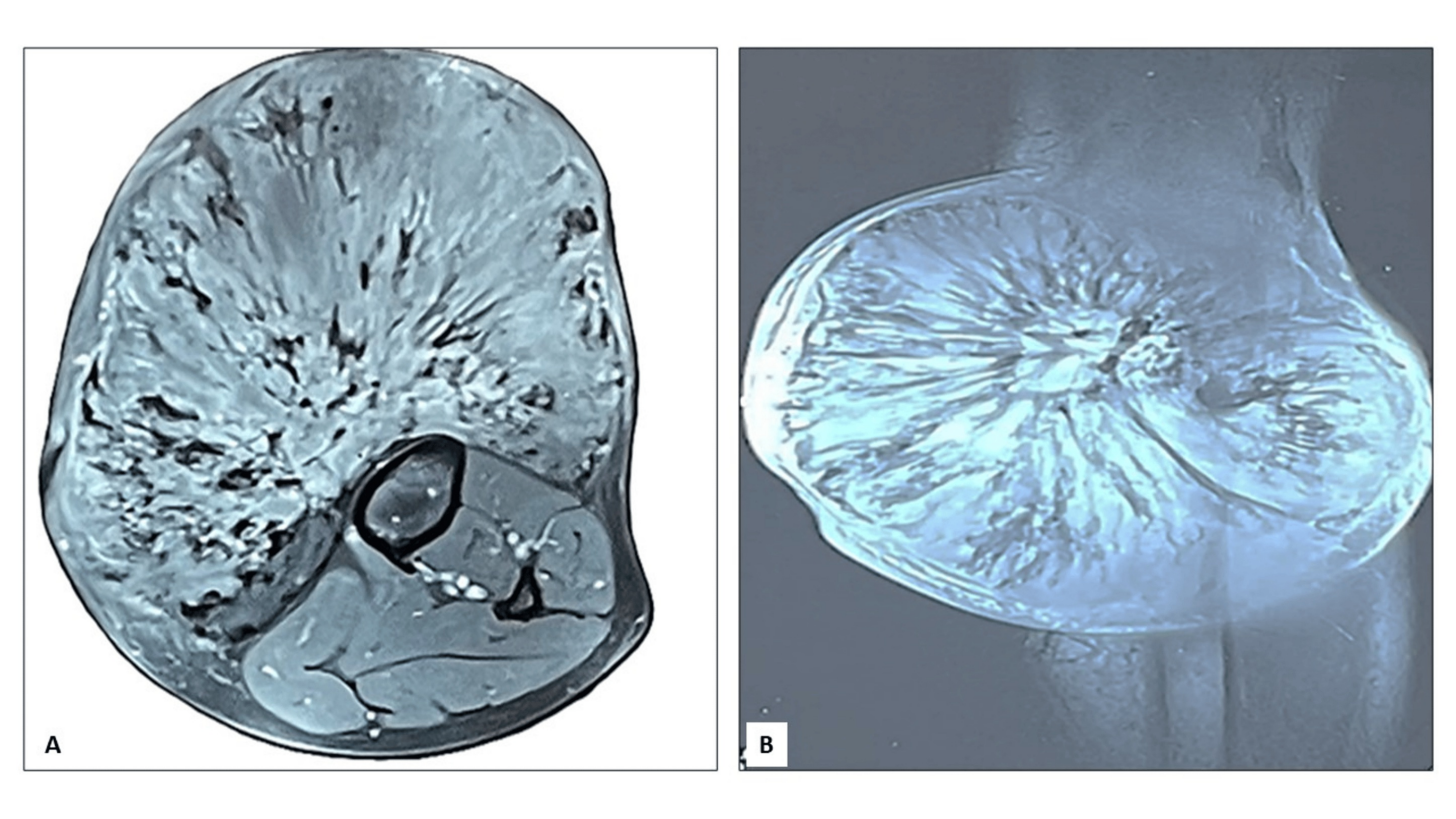
(A) Axial (T1) and (B) coronal (T2) MRI images showing soft tissue mass with bone involving the upper part of the tibia

Magnetic resonance imaging (MRI) revealed a 12.5 × 13.7 × 14.8 cm mass in its greatest dimensions located at the epiphyseal-diaphyseal junction with associated intramedullary involvement. The lesion exhibited an isointense signal on T1-weighted images and a hyperintense signal on T2-weighted sequences with focal hypointense areas on both T1 and T2 sequences, suggesting ossified or mineralized components (Figure 2).

**Figure 3:**
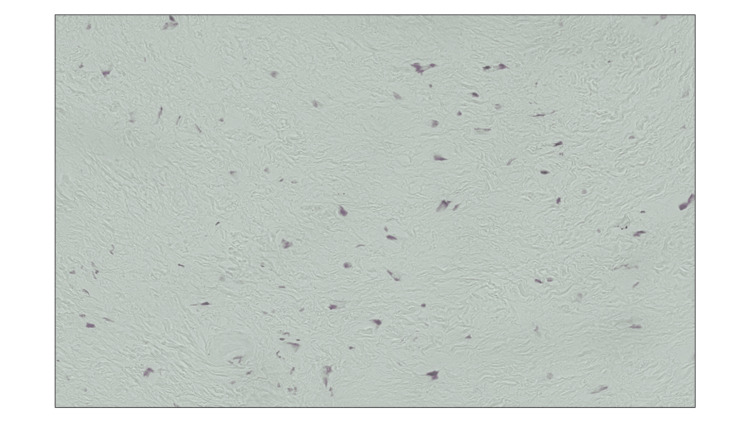
Clusters of atypical spindle cells with elongated, hyperchromatic nuclei embedded within a myxoid stroma (200X)

The first biopsy from the mass did not reveal any evidence of malignancy. Repeat core needle biopsy from the mass was done, and histopathological examination (HPE) revealed a spindle cell neoplasm showing an occasional pleomorphic cell exhibiting hyperchromatic and pleomorphic nuclei with a loose fibrocollagenous tissue with sparse cellularity consistent with a malignant spindle cell neoplasm due to the absence of any osteoid or bony trabeculae (Figure 3).

**Figure 4:**
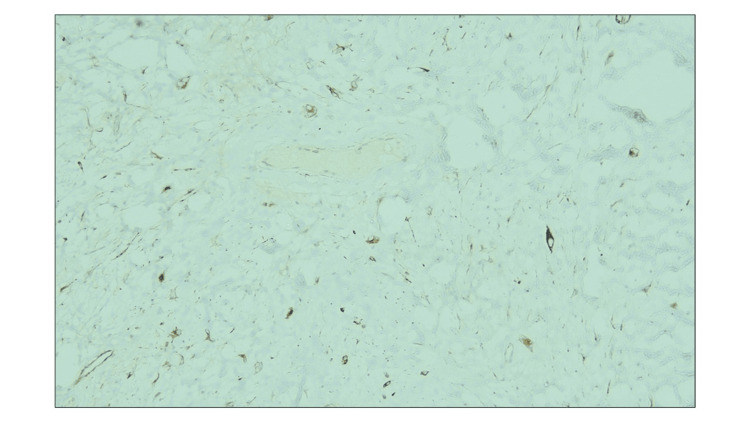
Atypical spindle cells exhibiting cytoplasmic positivity for desmin (100X)

On Immunohistochemistry (IHC) analysis, SMA was negative, and Bcl2 was non-specific in the biopsy sample. Desmin positivity was noted in tumour cells (Figure 4).

The final histopathology report of the biopsy from the tumour was reported as myxoid leiomyosarcoma. Due to a lack of osteoid or bone trabeculae in the biopsy specimen, the differential diagnosis of osteosarcoma did not feature in the initial biopsy. Although the X-ray and MRI features suggested the possibility of a bone tumor, possibly parosteal osteosarcoma, the paucicellular biopsy tissue sample limited our diagnosis. A metastatic workup, including computed tomography scans of the chest, abdomen, and pelvis, did not reveal any evidence of distant metastases.

**Figure 5:**
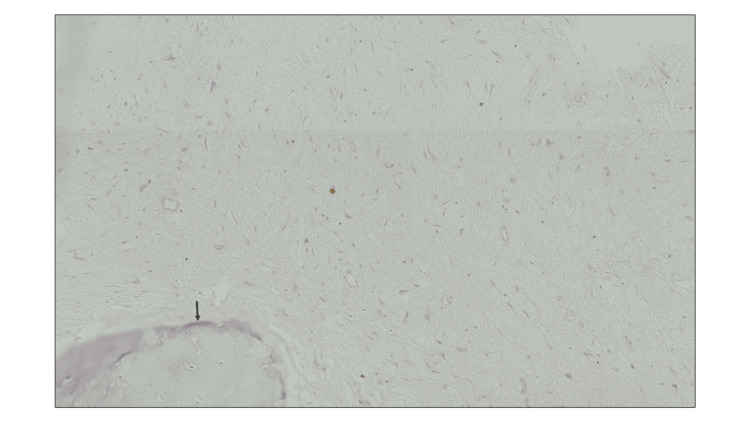
Scattered atypical cells with bony elements (arrow) (100X)

**Figure 6:**
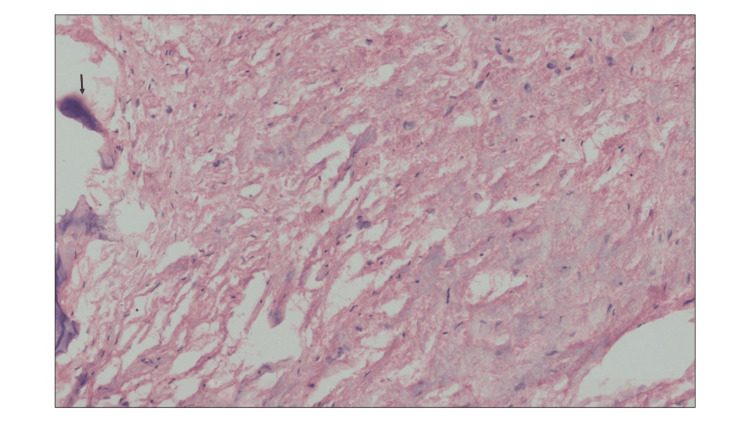
Spindle to oval neoplastic cells with an area of calcification (arrow) (200X)

The patient underwent surgery in January 2025 with an above-knee amputation procedure. Postoperative HPE revealed a 16x14.5x13 cm bony hard tumour. All cut margins were negative, and no neurovascular involvement was noted. Microscopically, there were loosely arranged sheets of spindle cells with vesicular chromatin, inconspicuous nucleoli, and abundant eosinophilic cytoplasm within a fibrocollagenous stroma showing areas of hyalinisation interspersed between bony trabeculae. There were collections of high-grade pleomorphic cells with pleomorphic hyperchromatic nuclei in focal areas (Figure 5, Figure 6).

**Figure 7:**
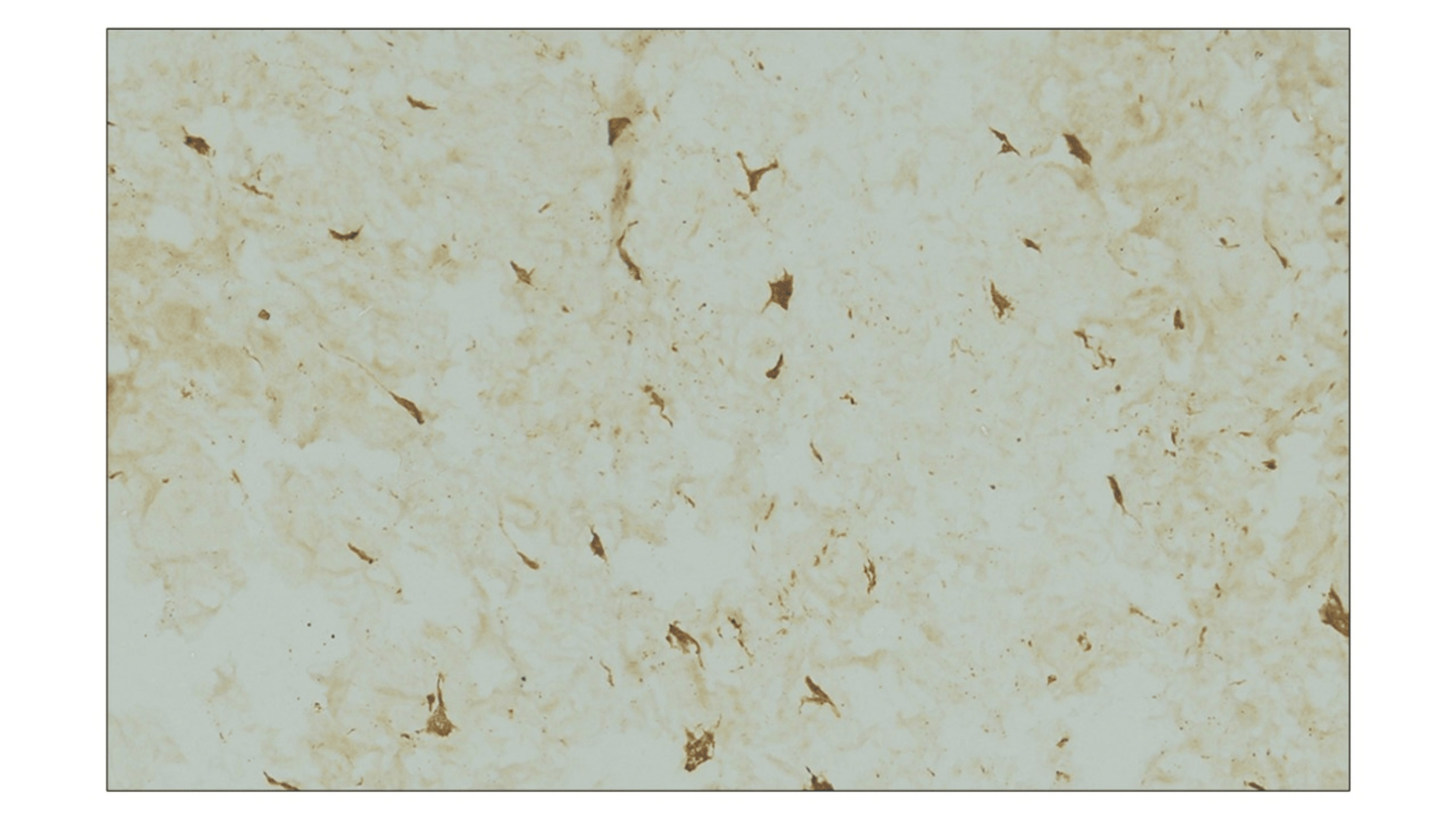
Atypical spindle cells demonstrating nuclear positivity for myogenin supporting rhabdomyosarcomatous differentiation (200X)

IHC analysis demonstrated positive staining for desmin and myogenin in the pleomorphic cells, while SMA and Bcl-2 were negative in the pleomorphic cells (Figure 7).

These radiological and histological features, along with immunohistochemistry, supported the diagnosis of parosteal osteosarcoma with rhabdomyosarcomatous dedifferentiation. Postoperatively, the patient was started on adjuvant chemotherapy in February 2025 with an ifosfamide and doxorubicin-based regimen after discussion in the tumour board. The patient took initial chemotherapy at our center and, in view of logistic issues, decided to continue treatment at a hospital near her hometown. The patient was lost to follow-up after three cycles of adjuvant chemotherapy.

## Discussion

Metastases from other primary sites account for the majority of bone cancer cases, particularly among the elderly. Bone sarcomas are extremely rare, accounting for only about 0.2% of all cancers. Osteosarcoma is the most common primary bone tumour, affecting mainly adolescents and young adults. The five-year overall survival rate is estimated to be approximately 67.9% [10]. The management protocol for osteosarcoma comprises neoadjuvant chemotherapy followed by limb-salvage surgery with adjuvant chemotherapeutic regimens adjusted and based on histopathological response assessment [11]. In our case, neoadjuvant chemotherapy was not given as limb salvage surgery was not considered due to an initial biopsy suggestive of myxoid leiomyosarcoma, which is considered inherently resistant to radiotherapy and chemotherapy. Also, as per the opinion of the surgical oncologist at our institute, limb salvage surgery was not considered feasible in view of the large size of the tumour.

Histologically, these tumours could dedifferentiate from primordial mesenchymal cells with the ability to differentiate into various cell types and may harbour different cell clones. Due to a lack of osteoid or bone trabeculae in the biopsy specimen, the differential diagnosis of osteosarcoma did not feature in the initial biopsy report. Also, a paucicellular biopsy tissue sample limited our diagnosis. However, correlating with radiological features and final surgical specimen featuring high-grade rhabdomyosarcomatous dedifferentiation with desmin and myogenin positivity along with scattered bony elements with occasional calcification, the diagnosis of parosteal osteosarcoma with rhabdomyosarcomatous features was confirmed. No specific markers for osteogenic sarcoma like Oct-4 (octamer-binding transcription factor 4) were used in our case, as the diagnosis of osteogenic sarcoma is made using the radiological features and the presence of osteoid.

Primary osteosarcoma of bone exhibiting rhabdomyosarcomatous differentiation is an extremely rare entity that indicates highly aggressive tumour biology and may have better prognosis [9]. Doxorubicin, cisplatin, and high-dose methotrexate form the backbone of osteosarcoma treatment, often combined with ifosfamide and etoposide, which also showed anti-tumour efficacy [12,13]. Dose adjustments or an alternative regimen may be necessary for older patients over 40 years of age, especially when methotrexate is administered at high doses [12]. High-dose adjuvant radiotherapy may be administered postoperatively to optimize local tumour control in cases with positive margins or unresectable diseases [14].

Reith et al. described a similar case of parosteal osteosarcoma with rhabdomyosarcomatous differentiation treated with surgery followed by adjuvant chemotherapy; however, the patient succumbed to extensive metastatic disease nine months following surgery [15]. Due to a lack of clinical data on osteosarcoma with rhabdomyosarcomatous differentiation, no established therapeutic protocol has been developed for this rare histological variant. The efficacy of chemotherapeutic intervention is unknown and requires further investigation.

## Conclusions

Primary osteosarcoma of the bone exhibiting rhabdomyosarcomatous differentiation is an exceptionally rare malignancy that poses diagnostic and therapeutic challenges due to its rarity and histopathological complexity. Whenever possible a larger biopsy sample may help in more tissue being available for definitive diagnosis with immunohistochemical profiling studies. Accurate diagnosis with biopsy may guide to consider neoadjuvant treatment of such tumours. Given the limited data available, no standardized treatment guidelines exist for this variant, highlighting the need for further case reports and clinical studies to improve understanding, guide therapy and evaluate prognosis.
